# The genome sequence of *Tenthredo amoena* Gravenhorst, 1807

**DOI:** 10.12688/wellcomeopenres.23298.1

**Published:** 2024-11-07

**Authors:** Steven Falk, Liam M. Crowley, Andrew Green, University of Oxford and Wytham Woods Genome Acquisition Lab, Darwin Tree of Life Barcoding collective, Wellcome Sanger Institute Tree of Life Management, Samples and Laboratory team, Wellcome Sanger Institute Scientific Operations: Sequencing Operations, Wellcome Sanger Institute Tree of Life Core Informatics team, Tree of Life Core Informatics collective, Darwin Tree of Life Consortium

**Affiliations:** 1 Independent researcher, Kenilworth, England, UK; 2 University of Oxford, Oxford, England, UK; 3 Sawfly Recording Scheme, Bedford, England, UK

**Keywords:** Tenthredo amoena, shiny-headed wasp-sawfly, genome sequence, chromosomal, Hymenoptera

## Abstract

We present a genome assembly from an individual female shiny-headed wasp-sawfly, *Tenthredo amoena* (Arthropoda; Insecta; Hymenoptera; Tenthredinidae). The genome sequence has a total length of 199.80 megabases. Most of the assembly (99.95%) is scaffolded into 18 chromosomal pseudomolecules. The mitochondrial genome has also been assembled and is 33.96 kilobases in length.

## Species taxonomy

Eukaryota; Opisthokonta; Metazoa; Eumetazoa; Bilateria; Protostomia; Ecdysozoa; Panarthropoda; Arthropoda; Mandibulata; Pancrustacea; Hexapoda; Insecta; Dicondylia; Pterygota; Neoptera; Endopterygota; Hymenoptera; Tenthredinoidea; Tenthredinidae; Tenthredininae; *Tenthredo*; *Tenthredo amoena* Gravenhorst, 1807 (NCBI:txid520940).

## Background

The *Tenthredo* genus has over one thousand species distributed across the Holarctic and Indomalayan regions. There are 30 species present in Britain. Within this genus, various species groups and subspecies have been identified. *Tenthredo amoena* Gravenhorst, 1807 falls within the subgenus *Zonuledo*, together in Britain with *Tenthredo distinguenda* (R. Stein, 1885), whose genome sequence has previously been published ( Falk *et al.*, 2023).

*Tenthredo amoena* is distributed across Europe. It is a black species, richly marked with yellow, and with a shiny head sparsely punctured around the frons and inner orbits. It is similar to *T. distinguenda* and *Tenthredo thompsoni* (Curtis, 1839), but in *amoena* the first antennal segment is entirely yellow, and the tegulae are marked with yellow on the front margin. In the female this is the only *Tenthredo* species in Britain where the fifth tergite is yellow at the base and black at the apex. The yellow tibiae are reddish-brown apically in the female but tipped with black in the male. Little is known about the ecology of the species, but many *Tenthredo* are predatory and contribute to pest control. The larvae feed on *Hypericum perforatum*, Perforate St John’s-wort, and *Hypericum maculatum*, Imperforate St John’s-wort, and as such are not considered a pest of agricultural or horticultural significance. The larvae are distinct from *T. distinguenda* which also feeds on St John’s-wort. The species is univoltine with adults on the wing from May to July.

The *Zonuledo* species are morphologically very similar, with high levels of intra-species character variability. Indeed, the genital structures of both males and females exhibit variability and are not considered reliable identification characteristics ( Taeger, 1991). Phylogenetic classification of the *Zonuledo* subgenus based on morphology is problematic due to the lack of obvious synapomorphic features. In BOLD, COI barcoding produces four well-defined clusters, namely *Tenthredo flavipennis* Brull, 1832, *Tenthredo zonula* Klug, 1817, *T. distinguenda* and *T. amoena*. All records of *T. amoena* fall within BIN AAK0580.

There are no previously barcoded specimens from Britain. Knowledge of sawfly evolution will benefit from the comparative analysis of genomes from closely and distantly related species. This female specimen from Wytham Woods, England matches the description of *T. amoena* using the characteristics in Benson’s key ( Benson, 1952) and the publication of the complete gene sequence will help our understanding of the phylogeny of this group.

## Genome sequence report

**Figure 1.**
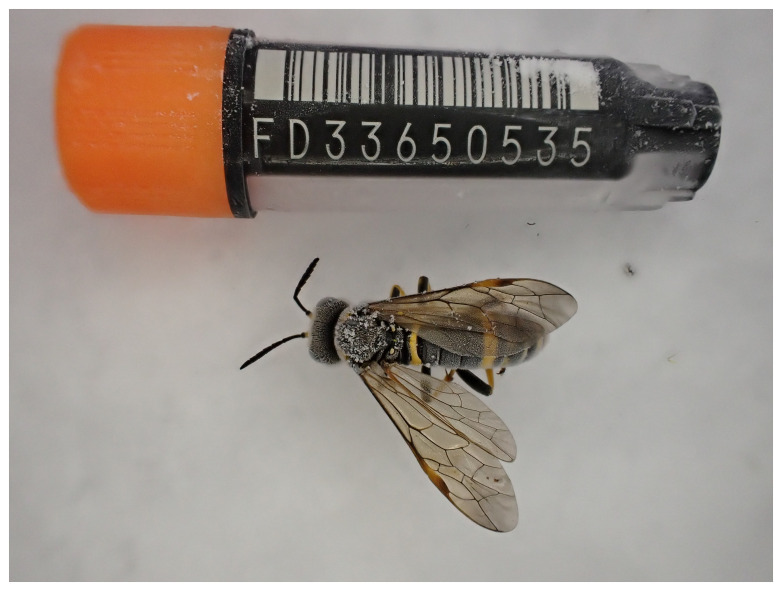
Photograph of the *Tenthredo amoena* (iyTenAmoe1) specimen used for genome sequencing.

The genome of an adult female specimen of *Tenthredo amoena* ( Figure 1) was sequenced using Pacific Biosciences single-molecule HiFi long reads, generating a total of 21.98 Gb (gigabases) from 2.18 million reads, providing approximately 111-fold coverage. Primary assembly contigs were scaffolded with chromosome conformation Hi-C data, which produced 90.42 Gb from 598.81 million reads. Specimen and sequencing details are provided in Table 1.

**Table 1.** Specimen and sequencing data for *Tenthredo amoena*.

Project information			
**Study title**	*Tenthredo amoena* (shiny-headed wasp-sawfly)		
**Umbrella BioProject**	PRJEB68254			
**Species**	*Tenthredo amoena*			
**BioSample**	SAMEA113425448			
**NCBI taxonomy ID**	520940			
Specimen information			
**Technology**	**ToLID**	**BioSample accession**	**Organism part**
**PacBio long read sequencing**	iyTenAmoe1	SAMEA113425545	Whole organism
**Hi-C sequencing**	iyTenAmoe1	SAMEA113425545	Whole organism
Sequencing information			
**Platform**	**Run accession**	**Read count**	**Base count (Gb)**
**Hi-C Illumina NovaSeq 6000**	ERR12259813	5.99e+08	90.42
**PacBio Revio**	ERR12257389	2.18e+06	21.98

Assembly errors were corrected by manual curation, including 43 missing joins or mis-joins. This reduced the scaffold number by 75.86% and increased the scaffold N50 by 2.31%. The final assembly has a total length of 199.80 Mb in 20 sequence scaffolds, with 99 gaps, and a scaffold N50 of 12.7 Mb ( Table 2).

**Table 2.** Genome assembly data for *Tenthredo amoena*, iyTenAmoe1.1.

Genome assembly		
Assembly name	iyTenAmoe1.1	
Assembly accession	GCA_963966615.1	
*Accession of alternate haplotype*	*GCA_963966555.1*	
Span (Mb)	199.80	
Number of contigs	120	
Number of scaffolds	20	
Longest scaffold (Mb)	24.28	
Assembly metrics *		*Benchmark*
Contig N50 length (Mb)	3.9	*≥ 1 Mb*
Scaffold N50 length (Mb)	12.7	*= chromosome N50*
Consensus quality (QV)	61.5	*≥ 40*
*k*-mer completeness	100.0%	*≥ 95%*
BUSCO **	C:95.7%[S:95.4%,D:0.3%], F:1.5%,M:2.8%,n:5,991	*S > 90%* *D < 5%*
Percentage of assembly mapped to chromosomes	99.95%	*≥ 90%*
Sex chromosomes	None	*localised homologous pairs*
Organelles	Mitochondrial genome: 33.96 kb	*complete single alleles*

* Assembly metric benchmarks are adapted from Rhie *et al.* (2021) and the Earth BioGenome Project Report on Assembly Standards September 2024. ** BUSCO scores based on the hymenoptera_odb10 BUSCO set using version 5.4.3. C = complete [S = single copy, D = duplicated], F = fragmented, M = missing, n = number of orthologues in comparison.

**Figure 2.**
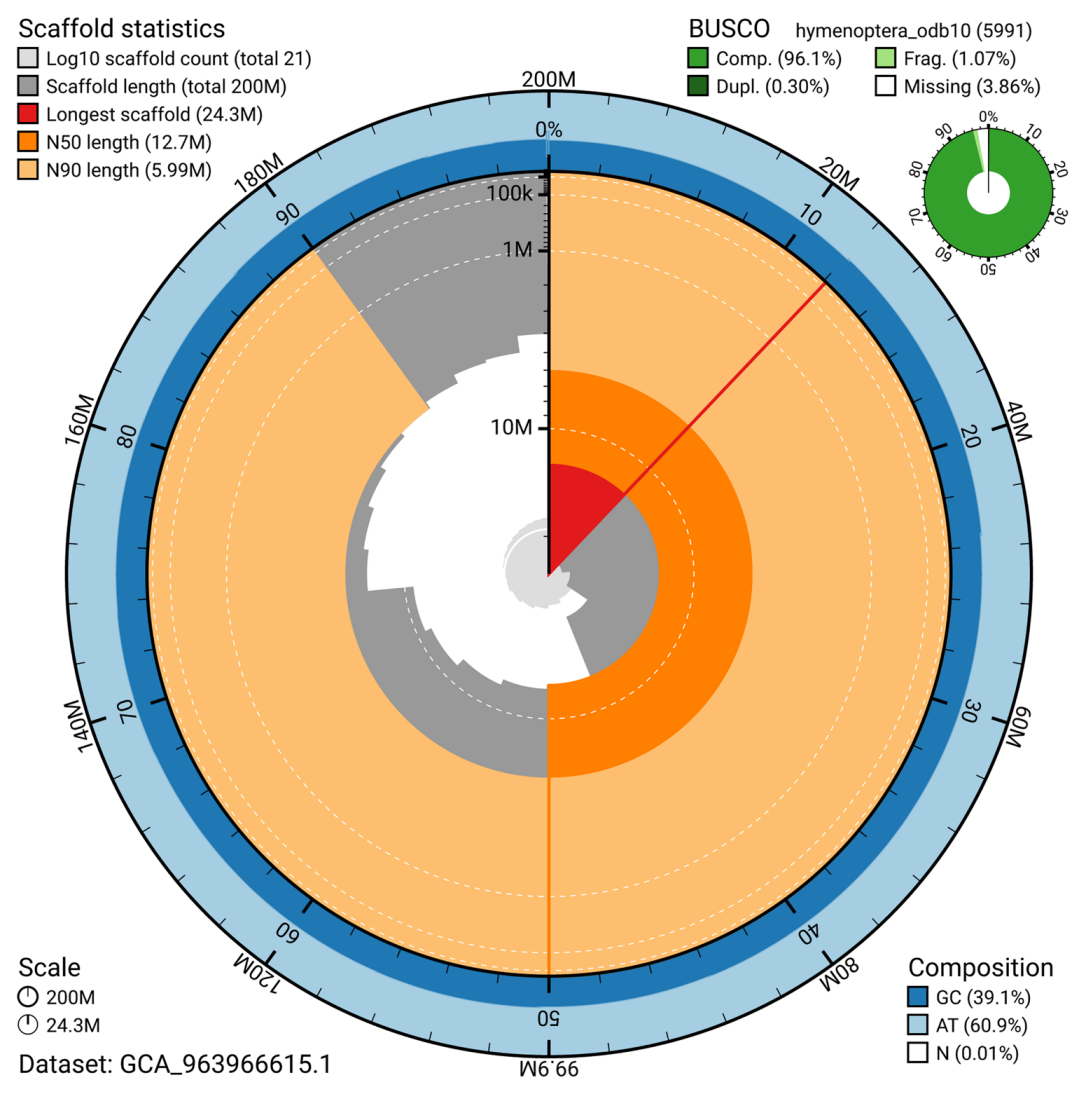
Genome assembly of *Tenthredo amoena*, iyTenAmoe1.1: metrics. The BlobToolKit snail plot shows N50 metrics and BUSCO gene completeness. The main plot is divided into 1,000 size-ordered bins around the circumference with each bin representing 0.1% of the 199,877,998 bp assembly. The distribution of scaffold lengths is shown in dark grey with the plot radius scaled to the longest scaffold present in the assembly (24,278,574 bp, shown in red). Orange and pale-orange arcs show the N50 and N90 scaffold lengths (12,736,242 and 5,991,294 bp), respectively. The pale grey spiral shows the cumulative scaffold count on a log scale with white scale lines showing successive orders of magnitude. The blue and pale-blue area around the outside of the plot shows the distribution of GC, AT and N percentages in the same bins as the inner plot. A summary of complete, fragmented, duplicated and missing BUSCO genes in the hymenoptera_odb10 set is shown in the top right. An interactive version of this figure is available at https://blobtoolkit.genomehubs.org/view/GCA_963966615.1/dataset/GCA_963966615.1/snail.

**Figure 3.**
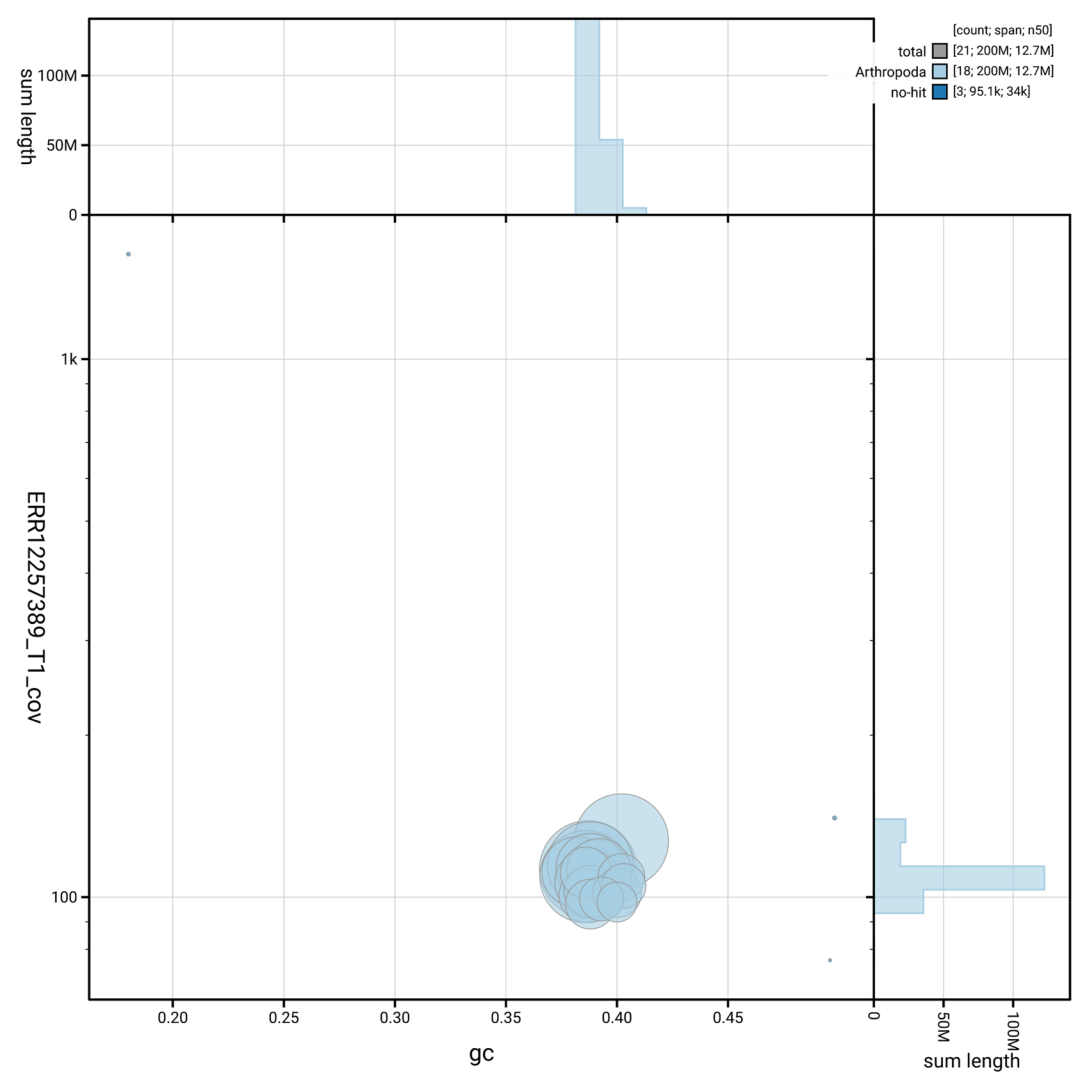
Genome assembly of *Tenthredo amoena:* Blot plot of base coverage in the raw data against GC proportion for sequences in iyTenAmoe1.1. Sequences are coloured by phylum. Circles are sized in proportion to sequence length. Histograms show the distribution of sequence length sum along each axis. An interactive version of this figure is available at https://blobtoolkit.genomehubs.org/view/GCA_963966615.1/dataset/GCA_963966615.1/blob.

**Figure 4.**
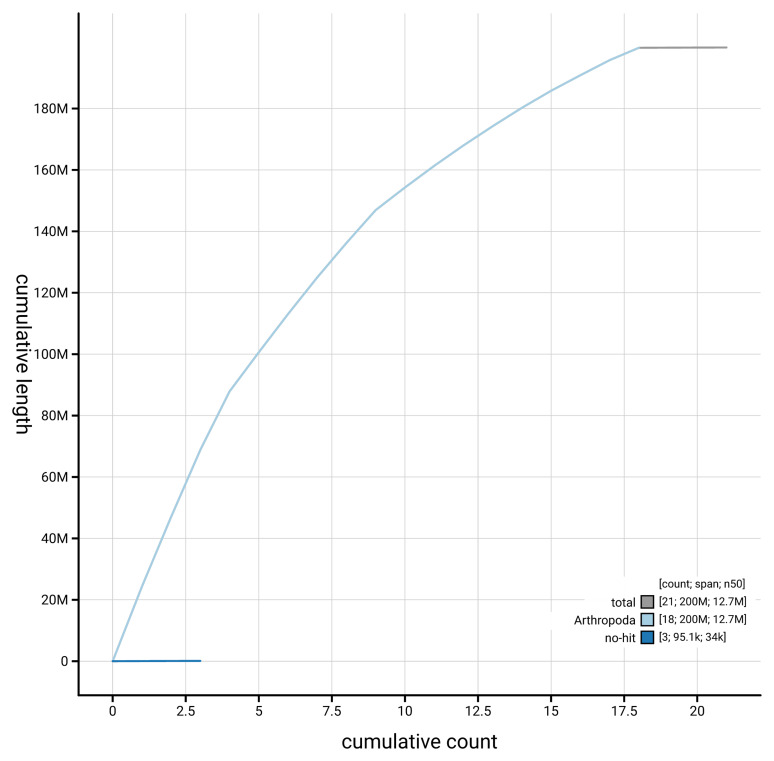
Genome assembly of *Tenthredo amoena* iyTenAmoe1.1: BlobToolKit cumulative sequence plot. The grey line shows cumulative length for all scaffolds. Coloured lines show cumulative lengths of scaffolds assigned to each phylum using the buscogenes taxrule. An interactive version of this figure is available at https://blobtoolkit.genomehubs.org/view/GCA_963966615.1/dataset/GCA_963966615.1/cumulative.

The snail plot in Figure 2 provides a summary of the assembly statistics, indicating the distribution of scaffold lengths and other assembly metrics. Figure 3 shows the distribution of scaffolds by GC proportion and coverage. Figure 4 presents a cumulative assembly plot, with separate curves representing different scaffold subsets assigned to various phyla, illustrating the completeness of the assembly.

**Figure 5.**
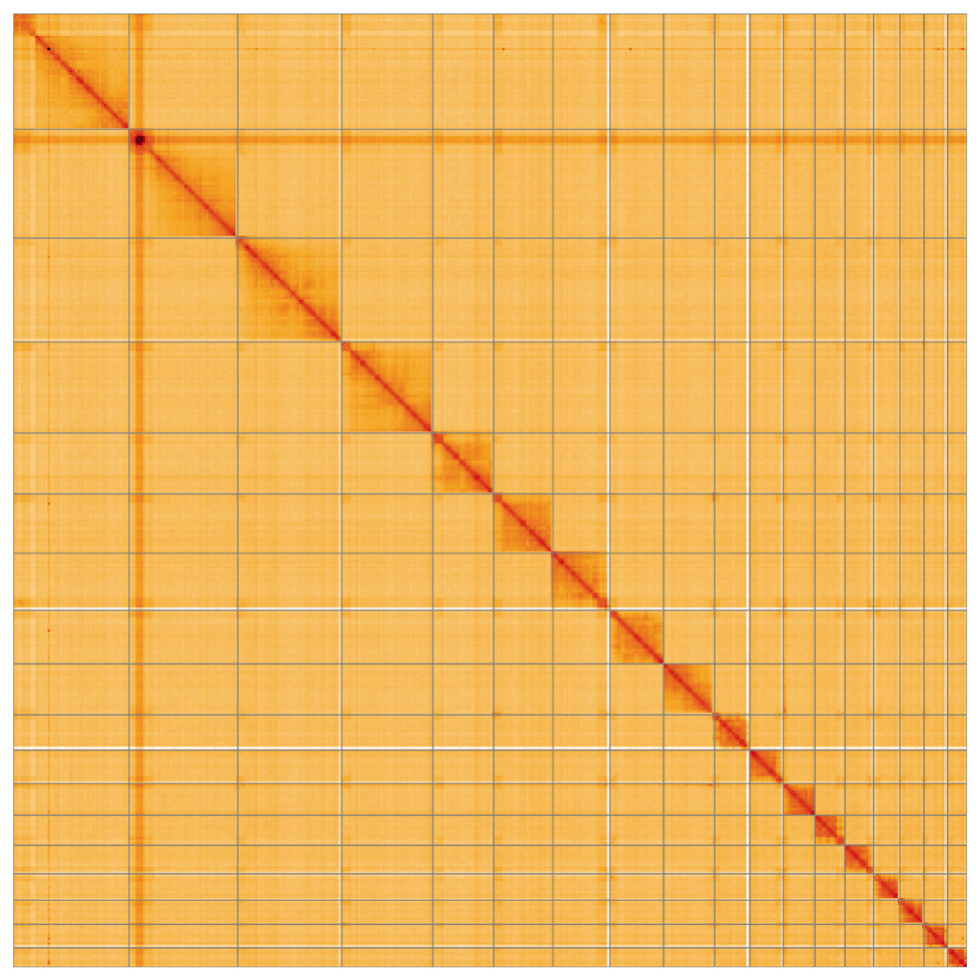
Genome assembly of *Tenthredo amoena* iyTenAmoe1.1: Hi-C contact map of the iyTenAmoe1.1 assembly, visualised using HiGlass. Chromosomes are shown in order of size from left to right and top to bottom. An interactive version of this figure may be viewed at https://genome-note-higlass.tol.sanger.ac.uk/l/?d=SZWQMfOWSeeJ2laCg-3x3w.

Most of the assembly sequence (99.95%) was assigned to 18 chromosomal-level scaffolds. Chromosome-scale scaffolds confirmed by the Hi-C data are named in order of size ( Figure 5; Table 3). During manual curation it was noted that the following regions of this assembly are of uncertain order and orientation: Chromosome 1 ~0–4.6 Mb, Chromosome 2 ~0–5.4 Mb, Chromosome 3 ~0–1.75 Mb, Chromosome 4 ~0–1.2 Mb, Chromosome 7 ~10.7 Mb to the end, Chromosome 13 ~5.9-End Mbp, Chromosome 14 ~4.8 Mb to the end, Chromosome 15 ~0–0.7 Mb, Chromosome 16 ~0–0.6 Mb.

**Table 3.** Chromosomal pseudomolecules in the genome assembly of *Tenthredo amoena*, iyTenAmoe1.

INSDC accession	Name	Length (Mb)	GC%
OZ016482.1	1	24.28	38.5
OZ016483.1	2	22.74	40.0
OZ016484.1	3	21.82	38.5
OZ016485.1	4	19.04	39.0
OZ016486.1	5	12.74	38.0
OZ016487.1	6	12.45	39.0
OZ016488.1	7	11.95	39.0
OZ016489.1	8	11.22	38.5
OZ016490.1	9	10.69	39.0
OZ016491.1	10	7.35	38.5
OZ016492.1	11	7.03	39.0
OZ016493.1	12	6.65	38.5
OZ016494.1	13	6.26	39.0
OZ016495.1	14	5.99	40.0
OZ016496.1	15	5.56	40.0
OZ016497.1	16	5.05	40.5
OZ016498.1	17	4.91	39.5
OZ016499.1	18	4.04	40.0
OZ016500.1	MT	0.03	18.0

While not fully phased, the assembly deposited is of one haplotype. Contigs corresponding to an alternate haplotype have also been deposited. The mitochondrial genome was also assembled and can be found as a contig within the multifasta file of the genome submission.

The estimated Quality Value (QV) of the final assembly is 61.5 with *k*-mer completeness of 100.0%, and the assembly has a BUSCO v5.4.3 completeness of 95.7% (single = 95.4%, duplicated = 0.3%), using the hymenoptera_odb10 reference set ( *n* = 5,991).

Metadata for specimens, BOLD barcode results, spectra estimates, sequencing runs, contaminants and pre-curation assembly statistics are given at https://links.tol.sanger.ac.uk/species/520940.

## Methods

### Sample acquisition and DNA barcoding

An adult female specimen of *Tenthredo amoena* (specimen ID Ox002812, ToLID iyTenAmoe1) was collected from Wytham Woods, Oxfordshire (biological vice-county Berkshire), United Kingdom (latitude 51.77, longitude –1.33) on 2022-07-14 by netting. The specimen was collected by Steven Falk (independent researcher) and Liam Crowley (University of Oxford), identified by Steven Falk and preserved on dry ice.

The initial identification was verified by an additional DNA barcoding process according to the framework developed by Twyford *et al*. (2024). A small sample was dissected from the specimens and stored in ethanol, while the remaining parts were shipped on dry ice to the Wellcome Sanger Institute (WSI). The tissue was lysed, the COI marker region was amplified by PCR, and amplicons were sequenced and compared to the BOLD database, confirming the species identification ( Crowley *et al.*, 2023). Following whole genome sequence generation, the relevant DNA barcode region was also used alongside the initial barcoding data for sample tracking at the WSI ( Twyford *et al.*, 2024). The standard operating procedures for Darwin Tree of Life barcoding have been deposited on protocols.io ( Beasley *et al.*, 2023).

### Nucleic acid extraction

The workflow for high molecular weight (HMW) DNA extraction at the Wellcome Sanger Institute (WSI) Tree of Life Core Laboratory includes a sequence of core procedures: sample preparation and homogenisation, DNA extraction, fragmentation and purification. Detailed protocols are available on protocols.io ( Denton *et al.*, 2023b). The iyTenAmoe1 sample was prepared for DNA extraction by weighing and dissecting it on dry ice ( Jay *et al.*, 2023), and tissue derived from the whole organism was homogenised using a PowerMasher II tissue disruptor ( Denton *et al.*, 2023a).

HMW DNA was extracted in the WSI Scientific Operations core using the Automated MagAttract v2 protocol ( Oatley *et al.*, 2023). The DNA was sheared into an average fragment size of 12–20 kb in a Megaruptor 3 system ( Bates *et al.*, 2023). Sheared DNA was purified by solid-phase reversible immobilisation, using AMPure PB beads to eliminate shorter fragments and concentrate the DNA ( Strickland *et al.*, 2023). The concentration of the sheared and purified DNA was assessed using a Nanodrop spectrophotometer and Qubit Fluorometer using the Qubit dsDNA High Sensitivity Assay kit. Fragment size distribution was evaluated by running the sample on the FemtoPulse system.

### Hi-C preparation

Tissue from the iyTenAmoe1 sample was processed at the WSI Scientific Operations core, using the Arima-HiC v2 kit. Frozen tissue (stored at –80 °C) was fixed, and the DNA crosslinked using a TC buffer with 22% formaldehyde. After crosslinking, the tissue was homogenised using the Diagnocine Power Masher-II and BioMasher-II tubes and pestles. Following the kit manufacturer's instructions, crosslinked DNA was digested using a restriction enzyme master mix. The 5’-overhangs were then filled in and labelled with biotinylated nucleotides and proximally ligated. An overnight incubation was carried out for enzymes to digest remaining proteins and for crosslinks to reverse. A clean up was performed with SPRIselect beads prior to library preparation.

### Library preparation and sequencing

Pacific Biosciences SMRTbell libraries were constructed using the Revio HiFi prep kit, according to the manufacturers’ instructions. DNA sequencing was performed by the Scientific Operations core at the WSI on a Pacific Biosciences Revio instrument.

For Hi-C library preparation, DNA was fragmented to a size of 400 to 600 bp using a Covaris E220 sonicator. The DNA was then enriched, barcoded, and amplified using the NEBNext Ultra II DNA Library Prep Kit following manufacturers’ instructions. The Hi-C sequencing was performed using paired-end sequencing with a read length of 150 bp on an Illumina NovaSeq 6000 instrument.

### Genome assembly, curation and evaluation

***Assembly***

The HiFi reads were first assembled using Hifiasm ( Cheng *et al.*, 2021) with the --primary option. Haplotypic duplications were identified and removed using purge_dups ( Guan *et al.*, 2020). The Hi-C reads were mapped to the primary contigs using bwa-mem2 ( Vasimuddin *et al.*, 2019). The contigs were further scaffolded using the provided Hi-C data ( Rao *et al.*, 2014) in YaHS ( Zhou *et al.*, 2023) using the --break option. The scaffolded assemblies were evaluated using Gfastats ( Formenti *et al.*, 2022), BUSCO ( Manni *et al.*, 2021) and MERQURY.FK ( Rhie *et al.*, 2020).

The mitochondrial genome was assembled using MitoHiFi ( Uliano-Silva *et al.*, 2023), which runs MitoFinder ( Allio *et al.*, 2020) and uses these annotations to select the final mitochondrial contig and to ensure the general quality of the sequence.

***Assembly curation***

The assembly was decontaminated using the Assembly Screen for Cobionts and Contaminants (ASCC) pipeline (article in preparation). Flat files and maps used in curation were generated in TreeVal ( Pointon *et al.*, 2023). Manual curation was primarily conducted using PretextView ( Harry, 2022), with additional insights provided by JBrowse2 ( Diesh *et al.*, 2023) and HiGlass ( Kerpedjiev *et al.*, 2018). Scaffolds were visually inspected and corrected as described by Howe *et al*. (2021). Any identified contamination, missed joins, and mis-joins were corrected, and duplicate sequences were tagged and removed. The curation process is documented at https://gitlab.com/wtsi-grit/rapid-curation (article in preparation).

***Evaluation of the final assembly***

The final assembly was post-processed and evaluated using the three Nextflow ( Di Tommaso *et al.*, 2017) DSL2 pipelines: sanger-tol/readmapping ( Surana *et al.*, 2023a), sanger-tol/genomenote ( Surana *et al.*, 2023b), and sanger-tol/blobtoolkit ( Muffato *et al.*, 2024). The readmapping pipeline aligns the Hi-C reads using bwa-mem2 ( Vasimuddin *et al.*, 2019) and combines the alignment files with SAMtools ( Danecek *et al.*, 2021). The genomenote pipeline converts the Hi-C alignments into a contact map using BEDTools ( Quinlan & Hall, 2010) and the Cooler tool suite ( Abdennur & Mirny, 2020). The contact map is visualised in HiGlass ( Kerpedjiev *et al.*, 2018). This pipeline also generates assembly statistics using the NCBI datasets report ( Sayers *et al.*, 2024), computes *k*-mer completeness and QV consensus quality values with FastK and MERQURY.FK, and runs BUSCO ( Manni *et al.*, 2021) to assess completeness.

The blobtoolkit pipeline is a Nextflow port of the previous Snakemake Blobtoolkit pipeline ( Challis *et al.*, 2020). It aligns the PacBio reads in SAMtools and minimap2 ( Li, 2018) and generates coverage tracks for regions of fixed size. In parallel, it queries the GoaT database ( Challis *et al.*, 2023) to identify all matching BUSCO lineages to run BUSCO ( Manni *et al.*, 2021). For the three domain-level BUSCO lineages, the pipeline aligns the BUSCO genes to the UniProt Reference Proteomes database ( Bateman *et al.*, 2023) with DIAMOND ( Buchfink *et al.*, 2021) blastp. The genome is also split into chunks according to the density of the BUSCO genes from the closest taxonomic lineage, and each chunk is aligned to the UniProt Reference Proteomes database with DIAMOND blastx. Genome sequences without a hit are chunked with seqtk and aligned to the NT database with blastn ( Altschul *et al.*, 1990). The blobtools suite combines all these outputs into a blobdir for visualisation.

The genome assembly and evaluation pipelines were developed using nf-core tooling ( Ewels *et al.*, 2020) and MultiQC ( Ewels *et al.*, 2016), relying on the Conda package manager, the Bioconda initiative ( Grüning *et al.*, 2018), the Biocontainers infrastructure ( da Veiga Leprevost *et al.*, 2017), as well as the Docker ( Merkel, 2014) and Singularity ( Kurtzer *et al.*, 2017) containerisation solutions.

Table 4 contains a list of relevant software tool versions and sources.

**Table 4.** Software tools: versions and sources.

Software tool	Version	Source
BEDTools	2.30.0	https://github.com/arq5x/bedtools2
BLAST	2.14.0	ftp://ftp.ncbi.nlm.nih.gov/blast/executables/blast+/
BlobToolKit	4.3.7	https://github.com/blobtoolkit/blobtoolkit
BUSCO	5.4.3 and 5.5.0	https://gitlab.com/ezlab/busco
bwa-mem2	2.2.1	https://github.com/bwa-mem2/bwa-mem2
Cooler	0.8.11	https://github.com/open2c/cooler
DIAMOND	2.1.8	https://github.com/bbuchfink/diamond
fasta_windows	0.2.4	https://github.com/tolkit/fasta_windows
FastK	427104ea91c78c3b8b8b49f1a7d6bbeaa869ba1c	https://github.com/thegenemyers/FASTK
Gfastats	1.3.6	https://github.com/vgl-hub/gfastats
GoaT CLI	0.2.5	https://github.com/genomehubs/goat-cli
Hifiasm	0.19.8-r587	https://github.com/chhylp123/hifiasm
HiGlass	44086069ee7d4d3f6f3f0012569789ec138f42b84aa44357 826c0b6753eb28de	https://github.com/higlass/higlass
Merqury.FK	d00d98157618f4e8d1a9190026b19b471055b22e	https://github.com/thegenemyers/MERQURY.FK
MitoHiFi	3	https://github.com/marcelauliano/MitoHiFi
MultiQC	1.14, 1.17, and 1.18	https://github.com/MultiQC/MultiQC
NCBI Datasets	15.12.0	https://github.com/ncbi/datasets
Nextflow	23.04.0-5857	https://github.com/nextflow-io/nextflow
PretextView	0.2	https://github.com/sanger-tol/PretextView
purge_dups	1.2.5	https://github.com/dfguan/purge_dups
samtools	1.16.1, 1.17, and 1.18	https://github.com/samtools/samtools
sanger-tol/ ascc	-	https://github.com/sanger-tol/ascc
sanger-tol/ genomenote	1.1.1	https://github.com/sanger-tol/genomenote
sanger-tol/ readmapping	1.2.1	https://github.com/sanger-tol/readmapping
Seqtk	1.3	https://github.com/lh3/seqtk
Singularity	3.9.0	https://github.com/sylabs/singularity
TreeVal	1.0.0	https://github.com/sanger-tol/treeval
YaHS	1.2a.2	https://github.com/c-zhou/yahs

### Wellcome Sanger Institute – Legal and Governance

The materials that have contributed to this genome note have been supplied by a Darwin Tree of Life Partner. The submission of materials by a Darwin Tree of Life Partner is subject to the **‘Darwin Tree of Life Project Sampling Code of Practice’**, which can be found in full on the Darwin Tree of Life website here. By agreeing with and signing up to the Sampling Code of Practice, the Darwin Tree of Life Partner agrees they will meet the legal and ethical requirements and standards set out within this document in respect of all samples acquired for, and supplied to, the Darwin Tree of Life Project.

Further, the Wellcome Sanger Institute employs a process whereby due diligence is carried out proportionate to the nature of the materials themselves, and the circumstances under which they have been/are to be collected and provided for use. The purpose of this is to address and mitigate any potential legal and/or ethical implications of receipt and use of the materials as part of the research project, and to ensure that in doing so we align with best practice wherever possible. The overarching areas of consideration are:

•     Ethical review of provenance and sourcing of the material

•     Legality of collection, transfer and use (national and international)

Each transfer of samples is further undertaken according to a Research Collaboration Agreement or Material Transfer Agreement entered into by the Darwin Tree of Life Partner, Genome Research Limited (operating as the Wellcome Sanger Institute), and in some circumstances other Darwin Tree of Life collaborators.

## Data Availability

European Nucleotide Archive: Tenthredo amoena (shiny-headed wasp-sawfly). Accession number PRJEB68254; https://identifiers.org/ena.embl/PRJEB68254. The genome sequence is released openly for reuse. The *Tenthredo amoena* genome sequencing initiative is part of the Darwin Tree of Life (DToL) project. All raw sequence data and the assembly have been deposited in INSDC databases. The genome will be annotated using available RNA-Seq data and presented through the Ensembl pipeline at the European Bioinformatics Institute. Raw data and assembly accession identifiers are reported in Table 1 and Table 2.

## Author information

Members of the University of Oxford and Wytham Woods Genome Acquisition Lab are listed here: https://doi.org/10.5281/zenodo.12157525.

Members of the Darwin Tree of Life Barcoding collective are listed here: https://doi.org/10.5281/zenodo.12158331.

Members of the Wellcome Sanger Institute Tree of Life Management, Samples and Laboratory team are listed here: https://doi.org/10.5281/zenodo.12162482.

Members of Wellcome Sanger Institute Scientific Operations: Sequencing Operations are listed here: https://doi.org/10.5281/zenodo.12165051.

Members of the Wellcome Sanger Institute Tree of Life Core Informatics team are listed here: https://doi.org/10.5281/zenodo.12160324.

Members of the Tree of Life Core Informatics collective are listed here: https://doi.org/10.5281/zenodo.12205391.

Members of the Darwin Tree of Life Consortium are listed here: https://doi.org/10.5281/zenodo.4783558.
