# Supplementary material for: The genome sequence of Tenthredo amoena Gravenhorst, 1807
Source: Wellcome Open Res. 2024 Nov 7;9:660. [Version 1] doi: 10.12688/wellcomeopenres.23298.1 (PMC11632218; doi:10.12688/wellcomeopenres.23298.1)
Supplement: Peer review history

# Reviewer report: Reviewer response for version 1

**DOI:** 10.21956/wellcomeopenres.25680.r112858

**Published:** 2024-12-12

**Authors:** Ruiqi Li

**Affiliations:** 1 University of Colorado Boulder, Boulder, Colorado, USA

Falk presents a high quality chromosome-level genome of *Tenthredo amoena. *While the genome itself and the genomic pipeline seems great, I have a minor suggestion on the introduction: The authors could expand what comparative analysis of genomes could be done for the future, and if there is any trait of interest worth digging into. For example, photosymbiosis in the marine cockles is a key trait that worth further investigation (Li et al. 2023 and Li et al. 2024) ^1,2,3^

Are sufficient details of methods and materials provided to allow replication by others?

Yes

Is the rationale for creating the dataset(s) clearly described?

Yes

Are the datasets clearly presented in a useable and accessible format?

Yes

Are the protocols appropriate and is the work technically sound?

Yes

Reviewer Expertise:

Genomics

I confirm that I have read this submission and believe that I have an appropriate level of expertise to confirm that it is of an acceptable scientific standard.

## References

[1] : Comparative transcriptomics revealed parallel evolution and innovation of photosymbiosis molecular mechanisms in a marine bivalve. *Proc Biol Sci* .2024;291(2023) : 10.1098/rspb.2023.2408 20232408 10.1098/rspb.2023.2408 38807516 PMC11285757

[2] : The genome sequence of the giant clam, Tridacna crocea (Lamarck, 1819). *Wellcome Open Research* .2023;8: 10.12688/wellcomeopenres.20580.1 10.12688/wellcomeopenres.20580.1

[3] : The genome sequence of the giant clam, Tridacna gigas (Linnaeus, 1758). *Wellcome Open Res* .2024;9: 10.12688/wellcomeopenres.21136.1 145 10.12688/wellcomeopenres.21136.1 38800516 PMC11116938

# Reviewer report: Reviewer response for version 1

**DOI:** 10.21956/wellcomeopenres.25680.r111780

**Published:** 2024-12-10

**Authors:** Doga Cedden

**Affiliations:** 1 University of Göttingen, Göttingen, Germany

The data note by Falk et al. reports a high quality genome assembly from *Tenthredo amoena. *The background information for the insect is adequate. The methodology for the genome assembly is sufficiently described and appropriate for the study. The authors also provide a detailed quality assessment of the assembled genome, with a k-mer completeness of 100% and a BUSCO score of  95.7%. I would agree with the other review regarding the assessment of the BUSCO score, i.e., it is a bit lower compared to other high-quality assemblies, but this likely stems from the hymenoptera_odb10 reference set rather than the assembly reported here, judging from the other quality assessments provided in the data note.

Are sufficient details of methods and materials provided to allow replication by others?

Yes

Is the rationale for creating the dataset(s) clearly described?

Yes

Are the datasets clearly presented in a useable and accessible format?

Yes

Are the protocols appropriate and is the work technically sound?

Yes

Reviewer Expertise:

I work on coleopteran pests using RNAi and RNA-seq.

I confirm that I have read this submission and believe that I have an appropriate level of expertise to confirm that it is of an acceptable scientific standard.

# Reviewer report: Reviewer response for version 1

**DOI:** 10.21956/wellcomeopenres.25680.r111783

**Published:** 2024-12-06

**Authors:** Marko Mutanen

**Affiliations:** 1 University of Oulu, Oulu, Finland

This paper published a genome sequence for a sawfly *Tenthredo amoena*. The species belongs to a group of taxonomically difficult species for which reason increased of availability of genomes is desirable, particularly as it seems that species delimitation of many groups will require a genomic insight due to the obscurity of morphological characters and common sharing of DNA barcode haplotypes between species. There is little doubt about the identification of this specimens as it was confirmed through barcoding which in this case seems to provide a safe identification. Also the voucher image supports the identification although it show critical parts only partly.

 To my best understanding, the results indicate a high quality of the genome as indicated by a relatively high BUSCO coverage (95.7%). This percentage is a bit lower I have seen in the case of most Lepidoptera, which probably reflects the features of the standard hymenopteran BUSCO set of loci rather than that many present loci were missed. Based on the provided plots, graphs and other data, I am convinced that the quality of the sequence is high.

Are sufficient details of methods and materials provided to allow replication by others?

Yes

Is the rationale for creating the dataset(s) clearly described?

Yes

Are the datasets clearly presented in a useable and accessible format?

Yes

Are the protocols appropriate and is the work technically sound?

Yes

Reviewer Expertise:

Molecular taxonomy, systematics and phylogenetics of insects

I confirm that I have read this submission and believe that I have an appropriate level of expertise to confirm that it is of an acceptable scientific standard.
